# Supplementary material for: Childhood maltreatment and suicidal ideation in Chinese children and adolescents: the mediation of resilience
Source: PeerJ. 2021 Jul 6;9:e11758. doi: 10.7717/peerj.11758 (PMC8269734; doi:10.7717/peerj.11758)
Supplement: Supplemental Information 2 [file peerj-09-11758-s002.docx]

| **A1 Gender** | |
| --- | --- |
| 1 | Boy |
| 2 | Girl |
| **A21 Ethnic** | |
| 1 | Han |
| 2 | Bai |
| 3 | Yi |
| 4 | Hui |
| 5 | Wa |
| 6 | Other |
| **A4 Residence** | |
| 1 | Township |
| 2 | Village |
| **A7 Grade** | |
| 1 | Primary school |
| 2 | Junior high school |
| 3 | Senior high school |
| **A8 Study style** | |
| 1 | Day students |
| 2 | Boarding students |
| **A9 If an only child** | |
| 1 | Yes |
| 2 | No |
| **A1121 Mother** | |
| 1 | Yes |
| 2 | No |
| **A1122 Live with father** | |
| 1 | Yes |
| 2 | No |
| **A1123 Live with grandparents** | |
| 1 | Yes |
| 2 | No |
| **A1124 Live with brothers and sisters** | |
| 1 | Yes |
| 2 | No |
| **A1125 Live with cousins** | |
| 1 | Yes |
| 2 | No |
| **A1126 Live with father's brothers or sisters** | |
| 1 | Yes |
| 2 | No |
| **A1127 Live with mother's brothers or sisters** | |
| 1 | Yes |
| 2 | No |
| **A1128 Live with others** | |
| 1 | Yes |
| 2 | No |
| **A121 Is your father alive？** | |
| 1 | Yes |
| 2 | No |
| **A1221 If you know your father’s age?** | |
| 1 | Yes |
| 2 | No |
| **A123 Father's education level** | |
| 0 | Unknown |
| 1 | Illiteracy and below |
| 2 | Elementary school |
| 3 | Junior high school |
| 4 | Senior high school |
| 5 | College and above |
| **A1241 Physical disability** | |
| 1 | Yes |
| 2 | No |
| **A1242 Mental illness** | |
| 1 | Yes |
| 2 | No |
| **A1243 Hypertension** | |
| 1 | Yes |
| 2 | No |
| **A1244 Diabetes** | |
| 1 | Yes |
| 2 | No |
| **A1245 Chronic hepatitis** | |
| 1 | Yes |
| 2 | No |
| **A1246 Tuberculosis** | |
| 1 | Yes |
| 2 | No |
| **A1247 Malignant tumor** | |
| 1 | Yes |
| 2 | No |
| **A1248 Coronary heart disease** | |
| 1 | Yes |
| 2 | No |
| **A1249 Pneumoconiosis/silicosis** | |
| 1 | Yes |
| 2 | No |
| **A12410 Arthritis** | |
| 1 | Yes |
| 2 | No |
| **A12411 Cerebrovascular disease and sequelae** | |
| 1 | Yes |
| 2 | No |
| **A12413 Cataract** | |
| 1 | Yes |
| 2 | No |
| **A12415 Other** | |
| 1 | Yes |
| 2 | No |
| **A125 Is your mother alive？** | |
| 1 | Yes |
| 2 | No |
| **A1261 If you know your mother’s age?** | |
| 1 | Yes |
| 2 | No |
| **A127 Mother's education level** | |
| 0 | Unknown |
| 1 | Illiteracy and below |
| 2 | Elementary school |
| 3 | Junior high school |
| 4 | Senior high school |
| 5 | College and above |
| **A1281 Physical disability** | |
| 1 | Yes |
| 2 | No |
| **A1282 Mental illness** | |
| 1 | Yes |
| 2 | No |
| **A1283 Hypertension** | |
| 1 | Yes |
| 2 | No |
| **A1284 Diabetes** | |
| 1 | Yes |
| 2 | No |
| **A1285 Chronic hepatitis** | |
| 1 | Yes |
| 2 | No |
| **A1286 Tuberculosis** | |
| 1 | Yes |
| 2 | No |
| **A1287 Malignant tumor** | |
| 1 | Yes |
| 2 | No |
| **A1288 Coronary heart disease** | |
| 1 | Yes |
| 2 | No |
| **A1289 Pneumoconiosis/silicosis** | |
| 1 | Yes |
| 2 | No |
| **A12810 Arthritis** | |
| 1 | Yes |
| 2 | No |
| **A12811 Cerebrovascular disease and sequelae** | |
| 1 | Yes |
| 2 | No |
| **A12813 Cataract** | |
| 1 | Yes |
| 2 | No |
| **A12815 Other** | |
| 1 | Yes |
| 2 | No |
