# Supplementary material for: Childhood maltreatment and suicidal ideation in Chinese children and adolescents: the mediation of resilience
Source: PeerJ. 2021 Jul 6;9:e11758. doi: 10.7717/peerj.11758 (PMC8269734; doi:10.7717/peerj.11758)
Supplement: Supplemental Information 3 [file peerj-09-11758-s003.doc]

**调查对象编号：**

**临沧市在校儿童/青少年心理健康调查**

| **姓 名：** |  |
| --- | --- |
| **联系电话：** |  |
| **家庭住址：** | **临沧市 县 　 乡镇（街道） 　 村（居委会）**  **（详细地址）** |

**审核日期： 年 月 日 质控员签名：**

**知情同意书**

亲爱的同学：

您好！

感谢您参加此次“儿童青少年心理健康调查”，这次调查的目的是了解您的心理健康状况，从而更有效地开展青少年心理问题的防治工作。内容包括您的一般情况、个人经历、行为习惯等问题。完成这个调查大概需要40分钟左右的时间。调查完成后我们将会为您提供评估结果供您参考。

我们会对您所提供的信息严格保密，不会向任何人（包括学校老师和同学）透露您的问卷信息。您所提供的信息将只提供给科学研究使用。在调查过程中，如您觉得不适，可选择随时终止调查。

感谢您的支持！

我经解释已明白本调查的有关事项并同意参与本调查

本人签字：

| **第一部分 基本信息** | | | | | | | | | | | | |
| --- | --- | --- | --- | --- | --- | --- | --- | --- | --- | --- | --- | --- |
| **A1 性别** | 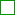 男 　　　　　　　 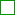 女 | | | | | | | | | | | |
| **A2 民族** | 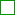 汉族 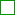 白族 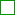 彝族 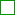 回族 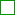 佤族  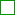 其它，请填写______________ | | | | | | | | | | | |
| **A3 出生日期** | ______ 年 ______ 月 ______日（公历/阳历日期） | | | | | | | | | | | |
| **A4 居住地** | 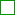 城镇 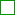 农村 | | | | | | | | | | | |
| **A5 现居住地址** | 请填写：____________________________ | | | | | | | | | | | |
| **A6 目前就读学校** | 请填写：____________________________ | | | | | | | | | | | |
| **A7 目前就读年级** | 请填写：____________________________ | | | | | | | | | | | |
| **A8 现就读方式** | 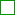 走读 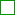 住校 | | | | | | | | | | | |
| **A9 是否独生子女** | 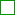 是 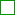 否 | | | | | | | | | | | |
| **A10 上小学起到现在的转学次数（升学不算）** | 请填写：____________________________ | | | | | | | | | | | |
| **A11 家庭常住成员** | **A11.1 目前和您长期居住的有______个人（不包括本人，一年中至少有6个月住一起）** | | | | | | | | | | | |
|  | **A11.2 和您长期居住的人是谁？（可多选）**  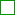母亲 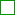父亲 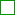祖父母 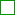亲兄弟姐妹 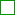堂/表兄弟姐妹 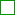父亲的兄弟姐妹 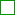母亲的兄弟姐妹 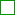其它_________________ | | | | | | | | | | | |
| **A12 父母基本情况** | **A12.1 您父亲目前是否健在？**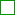 是 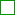 否（父亲去世时您_____周岁，*跳至12.5*） | | | | | | | | | | | |
|  | **A12.2 您父亲目前的年龄是________周岁** 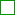 不知道 | | | | | | | | | | | |
|  | **A12.3 您父亲的文化程度是：**  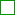文盲或文盲 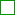小学 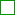初中  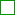高中/技校/中专 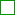大专及以上 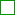 不详 | | | | | | | | | | | |
|  | **A12.4 您父亲目前是否患有以下疾病？（可多选）**  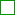肢体残疾 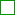精神疾病 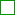高血压 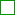糖尿病 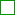慢性肝炎 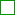肺结核 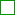恶性肿瘤 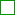冠心病 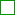尘肺/矽肺 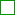关节炎 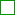脑血管疾病及后遗症 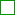白内障 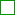其他____________________ 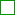无任何疾病 | | | | | | | | | | | |
|  | **A12.5 您母亲目前是否健在？**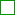 是 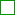 否（母亲去世时您_____周岁，*跳至13.1*） | | | | | | | | | | | |
|  | **A12.6 您母亲目前的年龄是________周岁** 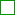 不知道 | | | | | | | | | | | |
|  | **A12.7您母亲的文化程度是：**  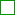文盲或文盲 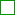小学 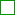初中  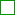高中/技校/中专 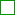大专及以上 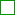 不详 | | | | | | | | | | | |
|  | **A12.8 您母亲目前是否患有以下疾病？（可多选）**  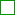肢体残疾 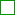精神疾病 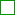高血压 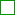糖尿病 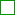慢性肝炎 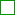肺结核 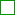恶性肿瘤 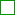冠心病 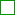尘肺/矽肺 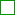关节炎 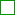脑血管疾病及后遗症 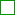白内障 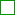其他_____________ 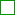无任何疾病 | | | | | | | | | | | |
|  | **A12.9 您父母的婚姻状况**  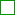在婚 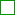离异 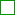再婚 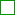丧偶 | | | | | | | | | | | |
|  | **A12.10 若父母再婚请继续填写以下问题**  **父母离异时您________周岁**  **父亲再婚时您________周岁或母亲再婚时您________周岁** | | | | | | | | | | | |
| **A13 家庭经济情况** | **A13.1 家庭收入来源**  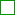家中有稳定的收入来源 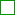需要靠低保或他人帮助 | | | | | | | | | | | |
|  | | | | | | | | | | | | |
| **第二部分 个人行为及经历** | | | | | | | | | | | | |
| *指导语：下面我们将询问您儿童期（16岁以前）的成长经历。请根据你的体会在每道题后面最适合你情况的选项下打“√”。* | | | | | | | | | | | | |
| **在我成长的过程中下列情况出现的频率** | | | 从不 | | 偶尔 | | 有时 | | 常常 | | 总是 | |
| **D1 当时家里没人关心我的饥饱。** | | |  | |  | |  | |  | |  | |
| **D2 当时有人照顾我、保护我。** | | |  | |  | |  | |  | |  | |
| **D3 当时家里有人喊我“笨蛋”“懒虫”或“丑八怪”等。** | | |  | |  | |  | |  | |  | |
| **D4 当时父母因为酗酒、吸毒或赌博以致不能照顾家庭。** | | |  | |  | |  | |  | |  | |
| **D5 当时家里有人使我觉得自己很重要或不一般。** | | |  | |  | |  | |  | |  | |
| **D6 当时家里没有人管我的衣着冷暖。** | | |  | |  | |  | |  | |  | |
| **D7 当时我感到家里人爱我。** | | |  | |  | |  | |  | |  | |
| **D8 当时我觉得父母希望从来没有生过我。** | | |  | |  | |  | |  | |  | |
| **D9 当时家里有人把我打伤的很重，不得不去医院。** | | |  | |  | |  | |  | |  | |
| **D10 当时我家的状况需要改善。** | | |  | |  | |  | |  | |  | |
| **D11 当时家里有人打的我皮肤青紫或留下疤痕。** | | |  | |  | |  | |  | |  | |
| **D12 当时家里有人用皮带、绳子、木板或其他硬东西惩罚我。** | | |  | |  | |  | |  | |  | |
| **D13 当时家里人彼此互相关心。** | | |  | |  | |  | |  | |  | |
| **D14 当时家里有人向我说过刻薄或侮辱性让我伤心的话。** | | |  | |  | |  | |  | |  | |
| **D15 我觉得我当时受到了躯体虐待。** | | |  | |  | |  | |  | |  | |
| **D16 我的童年是美好的。** | | |  | |  | |  | |  | |  | |
| **D17 当时我被打的很重，引起了老师、邻居或医生等的注意。** | | |  | |  | |  | |  | |  | |
| **D18 当时我觉得家里有人憎恨我。** | | |  | |  | |  | |  | |  | |
| **D19 当时家里人关系很亲密。** | | |  | |  | |  | |  | |  | |
| **D20 当时有人试图带有性色彩的方式触摸我或让我触摸他/她。** | | |  | |  | |  | |  | |  | |
| **D21 当时有人威逼或引诱我同他/她做性方面的事。** | | |  | |  | |  | |  | |  | |
| **D22 我觉得当时我的家好的不能再好了。** | | |  | |  | |  | |  | |  | |
| **D23 当时有人试图让我做或看性方面的事。** | | |  | |  | |  | |  | |  | |
| **D24 当时有人猥亵我，如耍流氓、动手动脚等。** | | |  | |  | |  | |  | |  | |
| **D25 当时我的心灵受到了折磨或虐待。** | | |  | |  | |  | |  | |  | |
| **D26 当时有人关心我的身体健康。** | | |  | |  | |  | |  | |  | |
| **D27 我当时受到了性虐待。** | | |  | |  | |  | |  | |  | |
| **D28 当时家是我获得力量和支持的源泉。** | | |  | |  | |  | |  | |  | |
| **D29第一次性行为时您多大年龄？** 岁（如从来没有填“88”） | | | | | | | | | | | | |
|  | | | | | | | | | | | | |
| ***指导语：下面将问您一些对于生命和死亡想法的问题，每个问题既问您最近一周是如何感觉的，又问您以前最消沉、心情最抑郁的时候是如何感觉的，请根据您的情况选择最合适的答案。*** | | | | | | | | | | | | |
| **I1 您希望活下去的程度如何？** | | | | | | | | | | | | |
| **I1.1 最近一周** | | 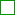中等到强烈 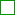弱 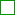没有活着的欲望 | | | | | | | | | | |
| **I1.2 以前最消沉、心情最抑郁的时候** | | 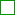中等到强烈 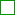弱 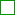没有活着的欲望 | | | | | | | | | | |
| **I2 您希望死去的程度如何？** | | | | | | | | | | | | |
| **I2.1 最近一周** | | 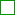没有死去的欲望 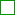弱 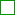中等到强烈 | | | | | | | | | | |
| **I2.2 以前最消沉、心情最抑郁的时候** | | 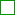没有死去的欲望 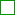弱 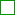中等到强烈 | | | | | | | | | | |
| **I3 您想要活下去的欲望超过想要死去的欲望吗？** | | | | | | | | | | | | |
| **I3.1 最近一周** | | 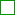活下去超过死去 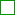差不多 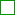死去超过活下去 | | | | | | | | | | |
| **I3.2 以前最消沉、心情最抑郁的时候** | | 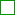活下去超过死去 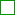差不多 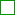死去超过活下去 | | | | | | | | | | |
| **I4 您主动尝试自杀的愿望程度如何？** | | | | | | | | | | | | |
| **I4.1 最近一周** | | 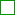没有 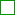弱 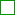中等到强烈 | | | | | | | | | | |
| **I4.2 以前最消沉、心情最抑郁的时候** | | 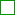没有 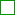弱 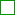中等到强烈 | | | | | | | | | | |
| **I5 您希望突然有外力来结束自己生命程度如何？（如睡着后不再醒来、遭遇车祸等意外死去等）** | | | | | | | | | | | | |
| **I5.1 最近一周** | | 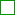没有 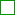弱 中等到强烈 | | | | | | | | | | |
| **I5.2 以前最消沉、心情最抑郁的时候** | | 没有 弱 中等到强烈 | | | | | | | | | | |
| ***指导语：下面有一些对于特定情况的描述，请在最符合您实际情况的选项处画√。*** | | | | | | | | | | | | |
|  | | | | **完全不符合** | | **比较不符合** | | **说不清** | | **比较符合** | | **完全符合** |
| **N1 失败总是让我感到气馁** | | | |  | |  | |  | |  | |  |
| **N2 我很难控制自己的不愉快情绪** | | | |  | |  | |  | |  | |  |
| **N3 我的生活有明确的目标** | | | |  | |  | |  | |  | |  |
| **N4 经历挫折后我一般会更加成熟有经验** | | | |  | |  | |  | |  | |  |
| **N5 失败和挫折会让我怀疑自己的能力** | | | |  | |  | |  | |  | |  |
| **N6 当我遇到不愉快的事情时，总找不到合适的倾述对象** | | | |  | |  | |  | |  | |  |
| **N7 我有一个或一些同龄朋友，可以将自己的困难讲给他/她听** | | | |  | |  | |  | |  | |  |
| **N8 父母很尊重我的意见** | | | |  | |  | |  | |  | |  |
| **N9 当我遇到困难需要帮助时，不知道该去找谁** | | | |  | |  | |  | |  | |  |
| **N10 我觉得与结果相比，事情的过程更能够帮助人成长** | | | |  | |  | |  | |  | |  |
| **N11 面临困难，我一般会定计划和解决方案** | | | |  | |  | |  | |  | |  |
| **N12 我习惯把事情憋在心里而不是向人倾诉** | | | |  | |  | |  | |  | |  |
| **N13 我认为逆境对人有激励作用** | | | |  | |  | |  | |  | |  |
| **N14 逆境有时候是对成长的一种帮助** | | | |  | |  | |  | |  | |  |
| **N15 父母总是喜欢干涉我的想法** | | | |  | |  | |  | |  | |  |
| **N16 在家里，我说什么总是没人听** | | | |  | |  | |  | |  | |  |
| **N17 父母对我缺乏信心和精神上的支持** | | | |  | |  | |  | |  | |  |
| **N18 我有困难的时候会主动找别人倾诉** | | | |  | |  | |  | |  | |  |
| **N19 父母从来不苛责我** | | | |  | |  | |  | |  | |  |
| **N20 面对困难时，我会集中自己的全部精力** | | | |  | |  | |  | |  | |  |
| **N21 我一般要过很久才能忘记不愉快的事情** | | | |  | |  | |  | |  | |  |
| **N22 父母总是鼓励我全力以赴** | | | |  | |  | |  | |  | |  |
| **N23 我能够很好的在短时间内调整情绪** | | | |  | |  | |  | |  | |  |
| **N24 我会为自己设定目标，以推动自己前进** | | | |  | |  | |  | |  | |  |
| **N25 我觉得任何事情都有其积极的一面** | | | |  | |  | |  | |  | |  |
| **N26 我心情不好也不愿意跟别人说** | | | |  | |  | |  | |  | |  |
| **N27 我情绪波动很大，容易大起大落** | | | |  | |  | |  | |  | |  |
|  | | | | | | | | | | | | |
| *所有调查到此结束！再次感谢您的配合！* | | | | | | | | | | | | |
