# Supplementary material for: Childhood maltreatment and suicidal ideation in Chinese children and adolescents: the mediation of resilience
Source: PeerJ. 2021 Jul 6;9:e11758. doi: 10.7717/peerj.11758 (PMC8269734; doi:10.7717/peerj.11758)
Supplement: Supplemental Information 4 [file peerj-09-11758-s004.doc]

**Participats code：**

**Investigation on mental health of children/adolescents in Lincang**

| **Name：** |  |
| --- | --- |
| **Cell：** |  |
| **Address：** |  |

**Date： Quality controller：**

**Informed Consent**

Dear students:

Hello!

Thank you for participating in the “Children and Adolescents Mental Health Survey”. The purpose of this survey is to understand your mental health status, in order to carry out the prevention and treatment on adolescent psychological issues more effectively. The content includes your general situation, personal experience, behavioral patterns, and other relevant questions. It will take you approximately 40 minutes to complete this survey. After finishing this survey, we will provide you with the evaluation results for your reference.

Your information will be strictly protected and will not be disclosed to anyone (including your teachers and classmates). The information you provide will be used only for scientific research. You can stop the investigation at any time if you have any discomfort during the process.

Thank you for your support!

I have understood relevant information of this survey after explanation and agreed to participate in this survey.

Signature:

| **Part 1 Gernaral Information** | | | | | | | | | | | | |
| --- | --- | --- | --- | --- | --- | --- | --- | --- | --- | --- | --- | --- |
| **A1 Gender** | 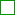 Boy 　　　　　　　 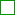 Girl | | | | | | | | | | | |
| **A2 Ethnic** | 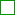 Han 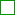 Bai 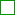 Yi 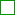 Hui 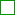 Wa  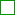 Other ______________ | | | | | | | | | | | |
| **A3 Date of birth** | _________________ | | | | | | | | | | | |
| **A4 Residence** | 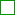 Township 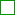 Village | | | | | | | | | | | |
| **A5 Current address** | ____________________________ | | | | | | | | | | | |
| **A6 Current school** | ____________________________ | | | | | | | | | | | |
| **A7 Grade** | ____________________________ | | | | | | | | | | | |
| **A8 Study style** | 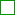 Day students 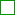 Boarding students | | | | | | | | | | | |
| **A9 If an only child** | 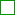 Yes 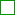 No | | | | | | | | | | | |
| **A10 Number of transfers** | ____________________________ | | | | | | | | | | | |
| **A11 Family members** | **A11.1 How many people are currently living with you for a long time? (not including yourself and living together for at least 6 months of the year)**___ | | | | | | | | | | | |
|  | **A11.2 Who have lived with you for a long time? (Multiple choice)**  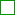Mother 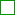Father 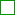Grandparents 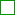Brothers and sisters 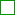Cousins 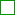Father's brothers or sisters 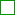Mother's brothers or sisters 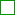Other_________________ | | | | | | | | | | | |
| **A12 Infoermation of parents** | **A12.1 Is your father alive？**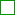 Yes 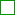 No (You were _____ years old when your father died, *to 12.5*) | | | | | | | | | | | |
|  | **A12.2 Father’s age ________** 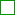 Unknown | | | | | | | | | | | |
|  | **A12.3 Father's education level：**  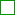Illiteracy and below 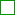Elementary school 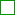Junior high school  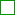Senior high school 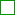College and above 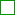 Unknown | | | | | | | | | | | |
|  | **A12.4 Does your father currently suffer from the following diseases? (Multiple choice)**  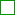Physical disability 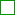Mental illness 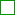Hypertension 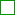Diabetes 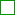Chronic hepatitis 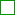Tuberculosis 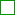Malignant tumor 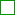Coronary heart disease 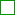Pneumoconiosis/silicosis 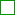Arthritis 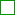Cerebrovascular disease and sequelae 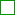Cataract 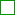Other____________________ 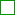No disease | | | | | | | | | | | |
|  | **A12.5 Is your mother alive？**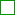 Yes 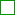 No (You were _____ years old when your mother died, *to 13.1*) | | | | | | | | | | | |
|  | **A12.6 Mother’s age ________** 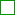 Unknown | | | | | | | | | | | |
|  | **A12.7 Mother's education level：**  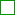Illiteracy and below 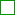Elementary school 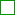Junior high school  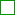Senior high school 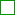College and above 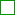 Unknown | | | | | | | | | | | |
|  | **A12.8 Does your father currently suffer from the following diseases? (Multiple choice)**  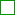Physical disability 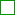Mental illness 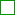Hypertension 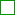Diabetes 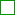Chronic hepatitis 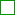Tuberculosis 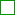Malignant tumor 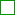Coronary heart disease 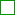Pneumoconiosis/silicosis 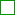Arthritis 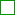Cerebrovascular disease and sequelae 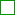Cataract 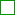Other____________________ 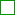No disease | | | | | | | | | | | |
|  | **A12.9 Marital status of the parents**  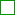Married 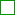Divorced 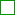Re-married 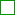Widowed | | | | | | | | | | | |
|  | **A12.10 Please continue to fill in the following questions if your parents are re-married**  **You were ________ years old when your parents divorced**  **You were ________ years old when your father remarried or you were ________ years old when your mother remarried** | | | | | | | | | | | |
| **A13 Family income** | **A13.1 Family income**  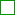A stable of family income 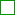An unstable of family income | | | | | | | | | | | |
|  | | | | | | | | | | | | |
| **Part 2 Personal experience** | | | | | | | | | | | | |
| *Guidance: Now we will ask you about your childhood (before 16 years of age) experience. Please tick “√” under the option that best suits your situation at the end of each question according to your experience.* | | | | | | | | | | | | |
| **How often did the following situations occur when I was growing up** | | | Never | | Rarely | | Sometimes | | often | | Very often | |
| **D1 My family did not care about my hunger and fullness.** | | |  | |  | |  | |  | |  | |
| **D2 Someone took care of me and protected me.** | | |  | |  | |  | |  | |  | |
| **D3 Someone in the family called me "stupid", "slacker" or "ugly guy".** | | |  | |  | |  | |  | |  | |
| **D4 My parents were unable to take care of the family because of alcohol, drug or gambling.** | | |  | |  | |  | |  | |  | |
| **D5 My family makes me feel important and indispensable.** | | |  | |  | |  | |  | |  | |
| **D6 No one cares about my clothes at home.** | | |  | |  | |  | |  | |  | |
| **D7 I felt that my family loved me.** | | |  | |  | |  | |  | |  | |
| **D8 I felt that my parents wished they never gave birth to me.** | | |  | |  | |  | |  | |  | |
| **D9 Someone in the family injured me badly and had to go to the hospital.** | | |  | |  | |  | |  | |  | |
| **D10 I felt that the condition of my family needed improvement.** | | |  | |  | |  | |  | |  | |
| **D11 I was bruised or scarred by someone at home.** | | |  | |  | |  | |  | |  | |
| **D12 Someone in the family punished me by belts, ropes, boards or other hard objects.** | | |  | |  | |  | |  | |  | |
| **D13 The family cared for each other.** | | |  | |  | |  | |  | |  | |
| **D14 Someone in the family insulted me and made me sad.** | | |  | |  | |  | |  | |  | |
| **D15 I think I was physically abused.** | | |  | |  | |  | |  | |  | |
| **D16 My childhood was wonderful.** | | |  | |  | |  | |  | |  | |
| **D17 I was beaten so badly that it attracted the attention of teachers, neighbors or doctors.** | | |  | |  | |  | |  | |  | |
| **D18 I felt that someone in my family hated me.** | | |  | |  | |  | |  | |  | |
| **D19 The family relationship was very close at the time.** | | |  | |  | |  | |  | |  | |
| **D20 Someone tried to touch me in a sexual way or let me touch him/her.** | | |  | |  | |  | |  | |  | |
| **D21 Someone threatened or tempted me to do sex with him/her.** | | |  | |  | |  | |  | |  | |
| **D22 I felt that my family could not be better.** | | |  | |  | |  | |  | |  | |
| **D23 Someone tried to get me to do or look at sexual things.** | | |  | |  | |  | |  | |  | |
| **D24 Someone molested me, such as playing a hooligan.** | | |  | |  | |  | |  | |  | |
| **D25 My mind was tortured or abused.** | | |  | |  | |  | |  | |  | |
| **D26 Someone cared about my health.** | | |  | |  | |  | |  | |  | |
| **D27 I was sexually abused.** | | |  | |  | |  | |  | |  | |
| **D28 My family was the source of strength and support.** | | |  | |  | |  | |  | |  | |
| **D29 How old were you at the first sexual behaviour?** （If it has never happened before, fill in "88"） | | | | | | | | | | | | |
|  | | | | | | | | | | | | |
| *Guidance: The following questions will ask you some thoughts about life and death. Each question asks how you felt during the past week and how you felt when you were most depressed. Please choose the most suitable answer depends on your situation.* | | | | | | | | | | | | |
| **I1 Desire to be alive.** | | | | | | | | | | | | |
| **I1.1 During the past week** | | 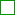Moderate to strong 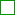Weak 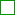No | | | | | | | | | | |
| **I1.2 When I was the most depressed** | | 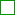Moderate to strong 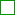Weak 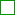No | | | | | | | | | | |
| **I2 Desire to death.** | | | | | | | | | | | | |
| **I2.1 During the past week** | | 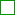No 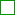Weak 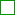Moderate to strong | | | | | | | | | | |
| **I2.2 When I was the most depressed** | | 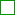No 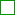Weak 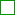Moderate to strong | | | | | | | | | | |
| **I3 Stronger desire to be alive or to death?** | | | | | | | | | | | | |
| **I3.1 During the past week** | | 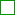Stronger desire to be alive 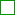equal 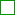Stronger desire to death | | | | | | | | | | |
| **I3.2 When I was the most depressed** | | 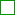Stronger desire to be alive 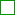equal 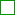Stronger desire to death | | | | | | | | | | |
| **I4 Desire to make active suicide attempt.** | | | | | | | | | | | | |
| **I4.1 During the past week** | | 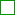No 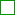Weak 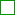Moderate to strong | | | | | | | | | | |
| **I4.2 When I was the most depressed** | | 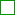No 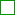Weak 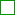Moderate to strong | | | | | | | | | | |
| **I5 Would avoid steps necessary to save or maintain life（Would leave life/death to chance）.** | | | | | | | | | | | | |
| **I5.1 During the past week** | | 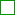No 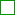Weak Moderate to strong | | | | | | | | | | |
| **I5.2 When I was the most depressed** | | No Weak Moderate to strong | | | | | | | | | | |
|  | | | | | | | | | | | | |
| *Guidance: Please choose the most suitable answer depends on your situation.* | | | | | | | | | | | | |
|  | | | | **Totally disagree** | | **Disagree** | | **Not sure** | | **Agree** | | **Totally agree** |
| **N1 Failure always makes me feel frustrated** | | | |  | |  | |  | |  | |  |
| **N2 I always feel hard to control my unpleasant emotion** | | | |  | |  | |  | |  | |  |
| **N3 My life has a clear purpose** | | | |  | |  | |  | |  | |  |
| **N4 After setback, usually I will be more sophisticated and more experienced** | | | |  | |  | |  | |  | |  |
| **N5 Failure and setback make me suspect my ability** | | | |  | |  | |  | |  | |  |
| **N6 When facing up unpleasant things, I always feel hard to find someone to talk to** | | | |  | |  | |  | |  | |  |
| **N7 I have a friend of my own age, whom I can talk my difficulties to** | | | |  | |  | |  | |  | |  |
| **N8 My parents respect my opinion** | | | |  | |  | |  | |  | |  |
| **N9 When in need, I don’t know whom I can reach to** | | | |  | |  | |  | |  | |  |
| **N10 Compared to result, I think process is more helpful to one’s growing up** | | | |  | |  | |  | |  | |  |
| **N11 When facing difficulties, usually I will make a plan** | | | |  | |  | |  | |  | |  |
| **N12 I’m used to put things inside, rather than telling someone else** | | | |  | |  | |  | |  | |  |
| **N13 I think adversity can be an inspiration** | | | |  | |  | |  | |  | |  |
| **N14 Sometimes adversity is helpful to one’s growing up** | | | |  | |  | |  | |  | |  |
| **N15 My parents like to interfere on my thoughts** | | | |  | |  | |  | |  | |  |
| **N16 In my home, they always pay no attention to my voice** | | | |  | |  | |  | |  | |  |
| **N17 My parents lack confidence and emotional support on me** | | | |  | |  | |  | |  | |  |
| **N18 When facing up difficulties, I will find someone to talk to** | | | |  | |  | |  | |  | |  |
| **N19 My parents never excoriate me** | | | |  | |  | |  | |  | |  |
| **N20 When facing difficulties, usually I will be fully concentrated** | | | |  | |  | |  | |  | |  |
| **N21 It usually takes a long time for me to get over unpleasant things** | | | |  | |  | |  | |  | |  |
| **N22 My parents always encourage me to do my best** | | | |  | |  | |  | |  | |  |
| **N23 I always can well adjust my emotion within a short period of time** | | | |  | |  | |  | |  | |  |
| **N24 I will set up a goal to push myself forward** | | | |  | |  | |  | |  | |  |
| **N25 I think everything has its positive side** | | | |  | |  | |  | |  | |  |
| **N26 I’m not willing to share my bad mood with someone else** | | | |  | |  | |  | |  | |  |
| **N27 My emotion fluctuates drastically** | | | |  | |  | |  | |  | |  |
|  | | | | | | | | | | | | |
| *It is the end of the investigation! Thanks for your cooperation!* | | | | | | | | | | | | |
